# *N‐*glycosylation Modification of CTSD Affects Liver Metastases in Colorectal Cancer

**DOI:** 10.1002/advs.202411740

**Published:** 2024-12-24

**Authors:** Nan Xiong, Yan Du, Chuncui Huang, Quanyi Yan, Long Zhao, Changjiang Yang, Qing Sun, Zhidong Gao, Caihong Wang, Jun Zhan, Hongquan Zhang, Shan Wang, Yingjiang Ye, Yan Li, Zhanlong Shen

**Affiliations:** ^1^ Department of Gastroenterological Surgery Peking University People's Hospital Beijing 100044 China; ^2^ Beijing Key Laboratory of Colorectal Cancer Diagnosis and Treatment Research Beijing 100044 China; ^3^ Laboratory of Surgical Oncology Peking University People's Hospital Beijing 100044 China; ^4^ Key Laboratory of Epigenetic Regulation and Intervention Institute of Biophysics Chinese Academy of Sciences 15 Datun Road Beijing 100101 China; ^5^ University of Chinese Academy of Sciences 19 Yuquan Road Beijing 100049 China; ^6^ Western Institute of Health Data Science 28 High Tech Avenue Chongqing 401329 China; ^7^ Program for Cancer and Cell Biology Department of Human Anatomy Histology and Embryology School of Basic Medical Sciences Peking University Health Science Center Beijing 100191 China

**Keywords:** cathepsin D, colorectal cancer, liver metastasis, N‐ glycosylation modification

## Abstract

Liver metastasis is the primary factor contributing to unfavorable prognosis in colorectal cancer (CRC). Although *N*‐glycosylation is implicated in metastasis, there is a notable paucity of comprehensive studies addressing the *N*‐glycosylation proteomics associated with liver metastasis in CRC. In this study, *N‐*glycosylated proteins and *N‐*glycosylation sites of differential expression between primary lesions and paired liver metastatic lesions are identified. Cathepsin D (CTSD) is further screened as a potentially pivotal *N‐*glycosylated protein in CRC liver metastasis. Glycosyltransferases complex DDOST and STT3B can regulate *N‐*glycosylation modification at residue 263 of CTSD (a protease), thereby affecting CTSD protease to lyse ACADM. ACADM can regulate ferroptosis‐related proteins (ACSL4, SLC7A11, and GPX4) to further influence the invasion and metastasis of CRC cells. This newly discovered mechanism provides potential therapeutic targets for CRC treatment and insights for controlling CRC progression and metastasis.

## Graphical abstract

The glycosyltransferase complex, comprising DDOST and STT3B, modulates the N‐glycosylation modification at residue 263 of cathepsin D (CTSD). The extent of N‐glycosylation at this specific residue influences CTSD's enzymatic capacity to hydrolyze acyl‐CoA dehydrogenase, medium chain (ACADM), thereby facilitating the liver metastasis of colorectal cancer.

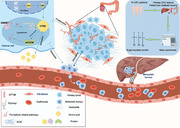

## 1 Introduction

Colorectal cancer (CRC) ranks among the top 5 deadliest cancers globally.^[^ ^1^ ^]^ In China, CRC has emerged as the second most common malignant cancer, surpassing rates in many developed countries and presenting a concerning trend.^[^ ^2^ ^]^ Despite advancements in the understanding, diagnosis, and treatment of CRC in recent years, the prognosis of patients with CRC remains unfavorable. Metastasis, particularly hepatic metastasis, is the primary factor contributing to the poor prognosis in these patients.^[^ ^3^ ^]^ 25% of patients with CRC have liver metastases at initial diagnosis and 15%–25% experience recurrence resulting from liver metastases after surgery.^[^ ^2^ ^]^ Furthermore, 90% of those with liver metastases cannot access effective treatment, resulting in a low 5‐year survival rate.^[^ ^2^ ^]^ For patients in IV stage (TNM), the 5‐year survival rate is just 24%.^[^ ^4^ ^]^ Therefore, elucidation of the mechanisms underlying liver metastasis in CRC is crucial for improving patient prognosis.

Glycosylation occurs in over 50% of natural proteins^[^ ^5^ ^]^ and plays key roles in protein folding, quality control, stability, transport, and function of proteins.^[^ ^6^ ^]^ Recent research has demonstrated that *N*‐glycosylation of glycoproteins plays a significant role in the metastasis of malignant tumors, promoting the disruption of cell adhesion and neovascularization as well as the activation of signal transduction pathways.^[^ ^7^ ^]^ The stability of the interferon‐gamma receptor protein was found to be reduced following *N‐*glycosylation modification, resulting in inhibition of the interferon‐gamma R signaling, thereby promoting immune evasion of CRC cells.^[^ ^8^ ^]^ A study demonstrated that the absence of *N‐*glycosylation modification at the Asn150 or Asn261 sites of the neuropilin‐1 protein can facilitate the invasion and metastasis of CRC cells.^[^ ^9^ ^]^

However, the aforementioned studies were scattered and single studies, which could not elucidate the key *N‐*glycosylated proteins involved in liver metastasis of CRC. Studies examining *N*‐glycosylated proteomics in CRC are limited. Research in this area remains in its nascent stages, particularly with regard to the differences in *N*‐glycosylated proteins between primary lesions and paired liver metastatic lesions. Elucidation of the *N*‐glycosylated proteomic profiles of primary and paired liver metastatic lesions will enhance our understanding of the molecular mechanisms underlying liver metastasis in CRC. This research is expected to provide substantial theoretical and practical value in identifying novel therapeutic targets for the treatment of liver metastases.

In this study, we aimed to analyze *N‐*glycosylated proteomic profiles in primary and paired liver metastatic lesions from CRC patients and to identify key factors involved in regulating metastases through *N‐*glycosylation modifications.

## 2 Results

### 2.1 *N‐*glycosylated Protein Profiling of Primary and Paired Liver Metastases Lesions from Patients with CRC

**Figure 1:**
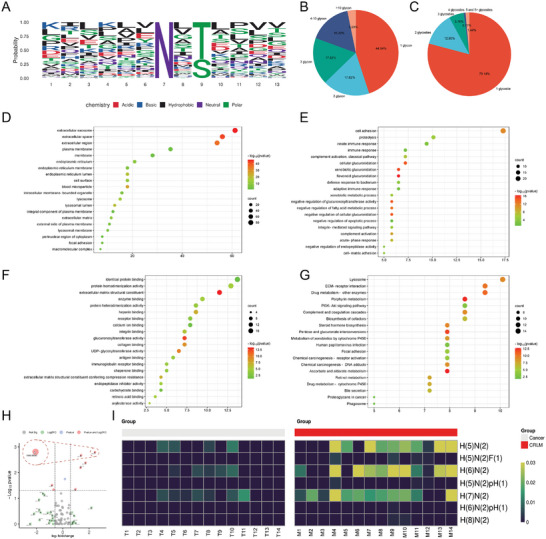
*N*‐glycosylated protein profiles of primary lesions and paired liver metastatic lesions. A) Conserved amino acid sequences surrounding all *N‐*glycosylation sites (N‐X‐S/T, X≠P). Specifically, the Asparagine (N) motif exhibits a relatively conserved nature; the terminal amino acid of Asparagine (N) can be any amino acid (X) with the exception of Proline (P); the penultimate residues are predominantly Serine (S) and Threonine (T). B) The ratio of the different numbers of glycosyls for each site. C) The proportion of *N‐*glycoproteins with the different numbers of *N‐*glycosylated modification sites. D−F) Gene Ontology enrichment analysis of cellular component, biological process analysis, and molecular function for these *N‐*glycosylated proteins (Fisher's exact test, *p* < .05). G) Kyoto Encyclopedia of Genes and Genomes enrichment analysis of potential pathways for these *N‐*glycosylated modification proteins (Fisher's exact test, *p* < .05). H) Volcano plot shows *N‐*glycosylation sites of differential expression. 2488.06545 (Precursor MH) exhibits the highest |fold change| of modification in liver metastatic lesions compared with primary lesions. I) The relative levels of various glycosyls in primary lesions and paired liver metastatic lesions.

To elucidate the molecular alterations of *N‐*glycosylated proteins involved in CRC metastasis, we compared the differences in *N‐*glycosylation modification between primary (*n * = 14) and paired liver metastatic (*n  *=  14) lesions using mass spectrometry. We detected 139 *N‐*glycosylated proteins, 185 *N‐*glycosylation modification sites, 490 *N‐*glycopeptides with entire structures, and 71 glycosyls (Table S1, Supplementary Table). Among these sites, 46 *N‐*glycosylation modification sites had not been reported by the Uniprot platform (Table S2, Supplementary Table). Compared with primary lesions, there were 13 sites in paired liver metastatic lesions that exhibited changes in *N‐*glycosylation modification levels exceeding 1.5‐fold (Table S3, Supplementary Table). **Figure** **1**A illustrates conserved amino acid sequences surrounding all *N‐*glycosylation sites. A total of 44.94% of *N‐*glycosylated sites exhibited modification by a singular, fixed glycosyl (Figure 1B), and 20.86% of *N‐*glycoproteins contained multiple *N‐*glycosylation modification sites (Figure 1C).

Gene Ontology (GO) and Kyoto Encyclopedia of Genes and Genomes analyses (KEGG) were used to investigate the potential functions of these *N‐*glycosylated proteins. Cellular Component analysis indicated that these *N‐*glycosylated proteins may belong to secretory and membrane proteins (Figure 1D) and were predominantly engaged in biological processes such as intercellular adhesion, proteolysis, and immune responses (Figure 1E), and primary molecular functions associated with binding activities (Figure 1F). KEGG analysis identified possible pathways related to *N‐*glycosylated proteins, such as lysosome, extracellular matrix‐receptor interaction, and drug‐receptor (Figure 1G).

2488.06545 (PrecursorMH) was found to be a structural modification of the H(6)N(2) glycosyl at residue 263 of Cathepsin D (CTSD) and it exhibited the highest |fold change| of modification in paired liver metastatic lesions relative to primary lesions (Figure 1H). *N‐*glycosylation modification levels of H(5)N(2) and H(7)N(2) were also significantly increased in paired liver metastatic lesions relative to primary lesions (Figure 1I). Consequently, paired liver metastatic lesions had higher *N‐*glycosylation modification (at residue 263 of CTSD) levels than primary lesions.

**Figure 2:**
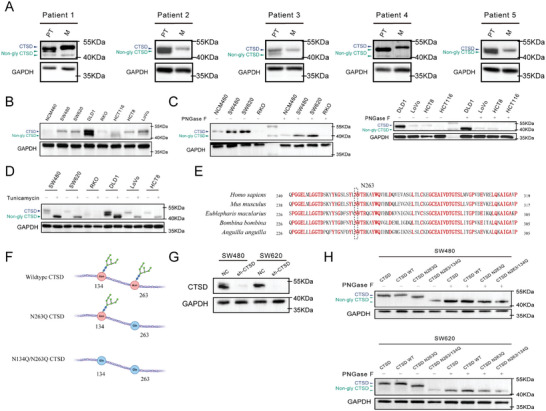
Verification of *N‐*glycosylated modification of CTSD. A) *N*‐glycosylation levels of CTSD in primary lesions and paired liver metastatic lesions from 5 patients with CRC. B) *N*‐glycosylation levels of CTSD in CRC cell lines. C, D) *N*‐glycosylation levels of CTSD in CRC cell lines following treatment with PNGase F and tunicamycin. E) Conserved sequences at the Asparagine residue 263 (N263) of CTSD for 5 species (*Homo sapiens*, *Mus musculus*, *Eublepharis macularius*, *Bombina bombina*, *Anguilla anguilla*). F) Asparagine was substituted with Glutamine (Asn → Gln) at residues 134 (N134Q) and 263 (N263Q) of CTSD. (G) CTSD was knocked down in SW480 and SW620 cell lines. H) Comparison of molecular weight of CTSD pre‐ and post‐PNGase F treatment.

*N‐*glycosylation modification levels were quantified by determining the molecular weights of CTSD. To confirm the presence of *N‐*glycosylation modifications on CTSD, we initially evaluated the glycosylation levels of CTSD in 5 patients with CRC. Paired liver metastatic lesions from patients with CRC had higher levels of *N‐*glycosylation modification on CTSD than primary lesions (**Figure** **2**A). In addition, we also evaluated the glycosylation levels of CTSD in CRC cell lines (Figure 2B). PNGase F is an amidase enzyme that cleaves *N‐*linked oligosaccharide chains from glycoproteins. Tunicamycin is a mixture of homologous nucleoside antibiotics that inhibit *N‐*linked glycosylation and block GlcNAc phosphotransferase (GPT). Following treatment with PNGase F (Yi Sheng, China) and Tunicamycin (MCE, USA), molecular weights of CTSD were found to decrease, indicating that CTSD had transitioned from a glycosylated form to a non‐glycosylated form (Figure 2C,D). These results indicated that CTSD exhibited *N‐*glycosylation modification, and paired liver metastatic lesions from patients with CRC demonstrated higher *N‐*glycosylation levels of CTSD than primary lesions.

### 2.2 Construction of Mutants for *N‐*glycosylation Modification Sites of CTSD

We downloaded *CTSD* RNA sequences for 5 species (*Homo sapiens*, *Mus musculus*, *Eublepharis macularius*, *Bombina bombina*, and *Anguilla anguilla*) from the NCBI database. The 5 species exhibited conserved evolution at the Asparagine residues 263 (N263) of CTSD (Figure 2E). The UniProt protein database predicted 2 potential *N*‐glycosylation sites, specifically at Asp residues 134 and 263, for CTSD. Given that Glutamine (Q) is the most analogous to Asparagine (N) in terms of physical and chemical properties, Asparagine was substituted with Glutamine (Asn → Gln, N263Q, N134Q) to minimize potential disruptions to other functions of CTSD (Figure 2F). Owing to the absence of low molecular weight (non‐glycosylated) bands and obvious N‐glycosylation modifications of CTSD in SW480 and SW620 cell lines (Figure 2B), the 2 cell lines were chosen for subsequent experimental investigations.

We silenced endogenous CTSD expression in SW480 and SW620 cell lines (Figure 2G). Then, these CTSD‐deficient cell lines were transduced with exogenous plasmids encoding wild‐type CTSD (CTSD WT) or mutant forms of CTSD (CTSD N263Q, CTSD N134Q/N263Q) (Figure 2H). This process yielded stable cell models that expressed CTSD WT, CTSD N263Q, and CTSD N134Q/N263Q. Figure 2H shows the presence of *N‐*glycosylation at residues 134 and 263 of CTSD. Furthermore, we observed a reduction in the molecular weight (owing to the transition from glycosylated to non‐glycosylated forms) of CTSD following mutations at the 2 sites (Figure 2H). CTSD N134Q/N263Q cells treated with PNGase F did not show alteration of molecular weight in CTSD; therefore, CTSD had only 2 *N‐*glycosylation modification sites at residues 134 and 263 (Figure 2H).

### 2.3 *N‐*glycosylation Modification of CTSD Affected Biological Processes in CRC Cells

In vitro experiments, invasion, proliferation, and anti‐apoptotic capacities of SW480 and SW620 cells were found to be significantly inhibited following a mutation at residue 134 and 263 of CTSD (Figure S1A− F, Supporting Information).

**Figure 3:**
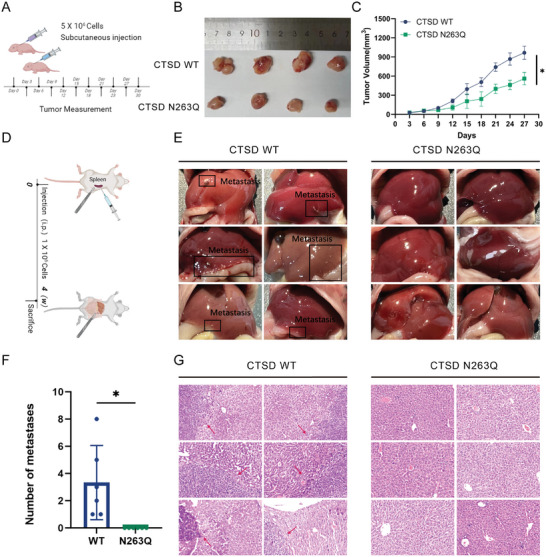
*N*‐glycosylation of CTSD affects biological processes in CRC cells in vivo. A) CTSD N263Q and CTSD WT cells were implanted into nude mice by subcutaneous injection. Tumor volume was measured at three‐day intervals. After a period of 4 weeks, nude mice were euthanized, and tumors were excised for photographic comparison. B) Macroscopic examination of tumors. CTSD N263Q cells exhibited reduced tumorigenic potential, forming significantly smaller tumors in comparison with CTSD WT cells. C) Subcutaneous tumor volume was quantified. The volume of tumors formed by CTSD N263Q cells was lower than that of tumors formed by CTSD WT cells (*n* = 4; *t*‐test; *p < .05; mean ± SEM; each group underwent 3 independent experiments). D) CTSD N263Q and CTSD WT cells were injected beneath the splenic capsule, and nude mice were euthanized after a period of 4 weeks. The presence of liver metastases in nude mice was initially assessed through gross examination, and metastases were subsequently confirmed via hematoxylin and eosin staining of tissue sections. E) Macroscopic examination of tumors. No liver metastasis was observed in the livers of CTSD N263Q nude mice, whereas metastasis was detected in the livers of CTSD WT nude mice. F) The number of metastasis tumors in livers. (*n *= 6; *t*‐test; *p < .05; mean ± SEM; each group underwent 3 independent experiments). G) Hematoxylin and eosin staining corroborated the presence of liver metastasis in CTSD WT nude mice, while no liver metastasis was observed in CTSD N263Q nude mice.

We constructed xenograft tumor models and liver metastasis models in nude mice by injecting CTSD WT and CTSD N263Q SW480 cells. In the xenograft tumor models, a mutation at residue 263 of CTSD (CTSD N263Q) significantly inhibited subcutaneous tumor growth in nude mice (**Figure** **3**A−C). In the liver metastasis model, no liver metastases were observed in CTSD N263Q nude mice, although metastases were present in the CTSD WT nude mic (Figure 3D−G). Specifically, the liver metastasis ability of SW480 cells was inhibited after *N‐*glycosylation modification levels of CTSD were down‐regulated (Figure 3E−G). These findings suggest that reducing *N‐*glycosylation modification levels at residue 263 of CTSD may inhibit CRC growth and metastasis.

### 2.4 *N*‐glycosylation Modification Alters the Folding Location, Stability, and Functional Activity of CTSD

**Figure 4:**
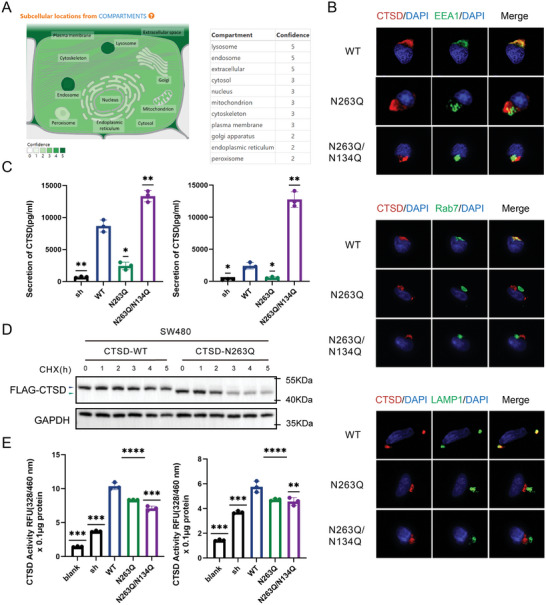
*N*‐glycosylation at residues 134 and 263 of CTSD regulated its folding location, stability, and functional activity. A)The intracellular and extracellular distribution of CTSD. CTSD was mainly localized in the extracellular matrix, lysosomes, and endosomes. B) Co‐localization of CTSD, lysosomes, and endosomes. CTSD failed to co‐localize with endosomal and lysosomal markers in both CTSD N263Q and CTSD N263Q/N134Q cell lines. (EEA1 for early endosomal marker, Rab7 for late endosomal marker, LAMP1 for lysosomal marker). C) In comparison with that in CTSD WT cells, secretion dynamics of CTSD N263Q cells decreased, while secretion dynamics of CTSD N263Q/N134Q cells increased (*n* = 3; *t*‐test; *p < .05, **p < .01; mean ± SEM; each group underwent 3 independent experiments). D) Stability analysis of CTSD. CTSD from CTSD N263Q cells exhibited significant degradation after 3 h of cycloheximide treatment, whereas CTSD WT did not show a significant decrease even after 5 h of cycloheximide treatment. E) CTSD enzyme activity analysis. The enzymatic activity of CTSD N263Q and CTSD N263Q/N134Q was observed to be lower than that of CTSD WT (*n* = 3; *t*‐test; **p < .01, ***p < .001 and ****p < .0001; mean ± SEM; each group underwent 3 independent experiments).

The GeneCards dataset^[^ ^10^ ^]^ showed that CTSD was mainly located in the extracellular matrix, lysosomes, and endosomes (**Figure** **4**A). Different organelles were labeled with specific markers (EEA1 for the early endosomal marker, Rab7 for the late endosomal marker, LAMP1 for the lysosomal marker). CTSD was unable to co‐localize with endosomal and lysosomal markers in CTSD N263Q and CTSD N263Q/N134Q cells (Figure 4B). In other words, down‐regulating *N‐*glycosylation modification levels at residue 263 of CTSD may inhibit its transport to endosomes and lysosomes in SW480 cells.

We also investigated the potential impact of *N‐*glycosylation modification at residue 263 of CTSD on its secretion dynamics. In comparison with CTSD WT cells, CTSD secretion of CTSD N263Q cells with the partial loss of *N‐*glycosylation modification decreased, while CTSD N263Q/N134Q cells with the absence of *N*‐glycosylation modification showed increased CTSD secretion (Figure 4C). Therefore, CRC cells could not secrete CTSD owing to the loss of *N*‐glycosylation modification of CTSD at residue 263.

Additionally, we used cycloheximide to study whether *N‐*glycosylation modification of CTSD at residue 263 affected its stability. Compared with that in CTSD WT cells, the quantity of CTSD was significantly reduced in CTSD N263Q cells after 3 h of cycloheximide treatment (Figure 4D), indicating that *N*‐glycosylation modification of CTSD at residue 263 affects its stability.

CTSD is an acid protease. We used the CTSD protease activity kit to detect CTSD N263Q and CTSD N263Q/N134Q enzyme activity. Fluorescence values were used to quantify the strength of enzyme activity. CTSD from CTSD N263Q cells and CTSD N263Q/N134Q cells had lower fluorescence values than that from CTSD WT cells (Figure 4E). Specifically, reducing *N‐*glycosylation modification levels at residues 134 and 263 of CTSD may decrease its protease activity (Figure 4E).

In conclusion, *N*‐glycosylation modification of CTSD at residues 134 and 263 may alter its folding location, stability, and functional activity.

### 2.5 STT3B and DDOST are *N‐*glycosyltransferases of CTSD

**Figure 5:**
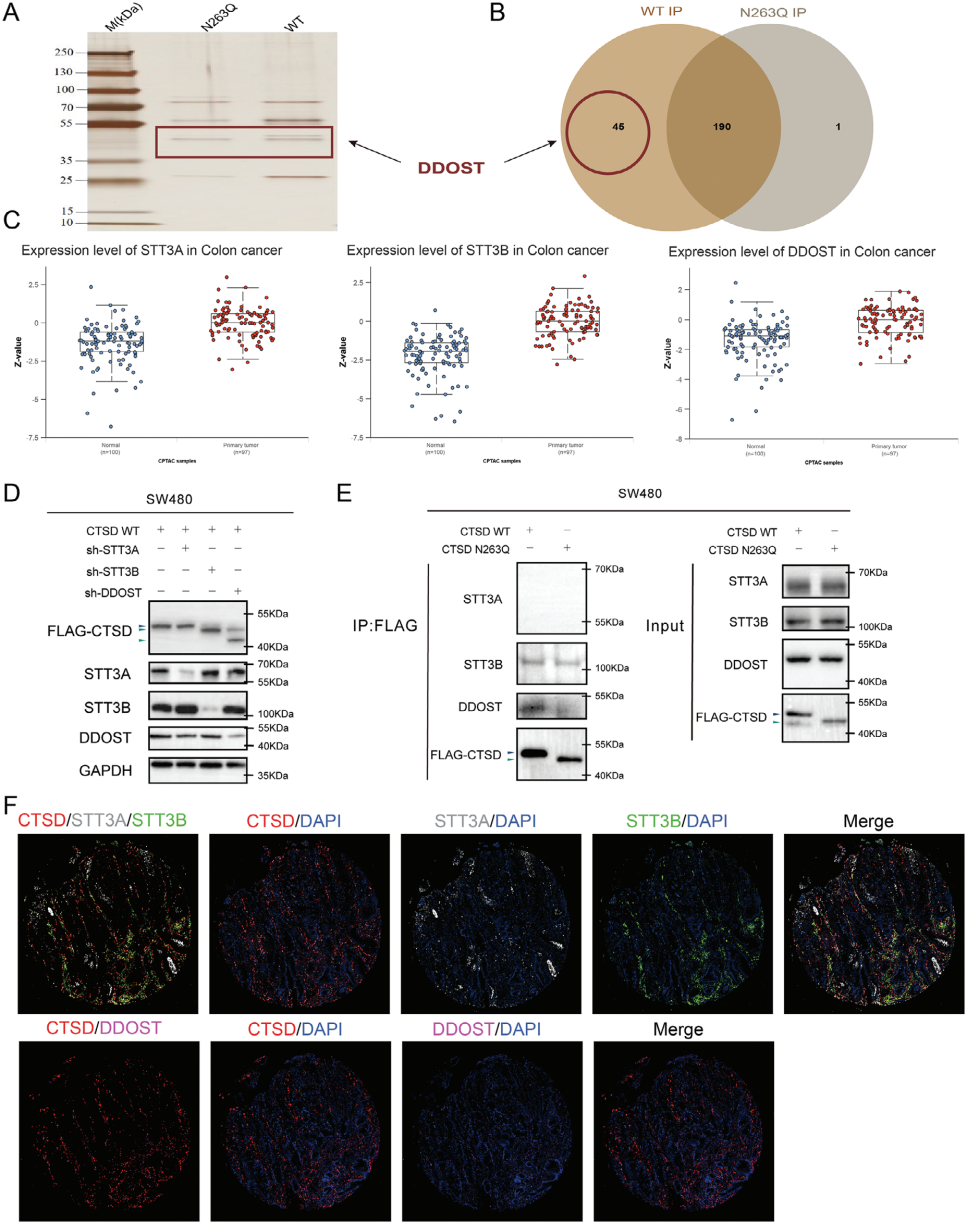
Identification of *N*‐glycosyltransferase for CTSD. A) CTSD with a FLAG label was enriched using FLAG magnetic beads and silver‐stained gel showed that proteins, such as DDOST, were enriched by CTSD. B) 191 proteins were enriched by CTSD N263Q and 235 proteins were enriched by CTSD WT, while 45 proteins exclusively interacted with CTSD WT. C) Expression levels of STT3A, STT3B, and DDOST were upregulated in colon cancer relative to normal tissues. Boxplots show median (central line), upper and lower quartiles (box limits), and 1.5 × interquartile range (whiskers). D) *N‐*glycosylated modification at residue 263 of CTSD was regulated by STT3B and DDOST. When STT3B and DDOST were knocked out, molecular weights of CTSD decreased significantly. E) Co‐immunoprecipitation and western blot analysis demonstrated that CTSD formed complexes with STT3B and DDOST. F) Immunofluorescence analysis illustrated the co‐localization of DDOST and STT3B with CTSD, whereas no such co‐localization was observed with STT3A.

We used co‐immunoprecipitation to enrich proteins binding with CTSD, and those proteins were subsequently identified by mass spectrometry analysis (**Figure** **5**A). The analysis results revealed that 235 proteins interacted with CTSD WT and 191 proteins combined with CTSD N263Q. Among the 45 proteins that exclusively interacted with CTSD WT, DDOST (also known as OST48) was the only *N‐*glycosyltransferase (Figure 5B) and may be involved in *N‐*glycosylation modification at residue 263. The initial *N‐*glycosyltransferase, also known as oligosaccharide transferase complex (OST),^[^ ^11^ ^]^ consisted of multiple proteins in a complex. OST existed in 2 forms, OST‐A and OST‐B, with STT3A and STT3B as their respective catalytic subunits, each responsible for initial *N‐*glycosylation. Catalytic subunits were capable of independently completing the *N‐*glycosylation addition process. However, in certain instances, various non‐catalytic subunits, such as DDOST, were required to form stable complexes with the substrate during the process.^[^ ^12^ ^]^ We used CPTAC, an online proteomic database, to investigate the expression levels of STT3A, STT3B, and DDOST between colon cancer tissues and paired normal tissues, and found that the 3 proteins were upregulated in colon cancer tissues (Figure 5C).

To investigate the effects of DDOST on *N‐*glycosylation modification at residue 263 and to identify the corresponding catalytic subunit, we constructed stable SW480 cell lines with targeted knockouts of STT3A, STT3B, and DDOST (Figure 5D). When STT3B and DDOST expression were reduced, the molecular weights of CTSD significantly decreased relative to the negative control, indicating a shift from *N‐*glycosylation to non‐*N‐*glycosylation (Figure 5D). These findings suggest that *N‐*glycosylation at residue 263 of CTSD is potentially regulated by DDOST, and STT3B likely served as the catalytic subunit at this site.

We used co‐immunoprecipitation and western blot to confirm CTSD combined with STT3B and DDOST (Figure 5E). We employed multiple immunofluorescence assays to determine the localization of CTSD, DDOST, STT3A, and STT3B in CRC tissues. Figure 5F demonstrated the co‐localization of DDOST and STT3B with CTSD, but not STT3A. These findings suggest that STT3B and DDOST are integral components of the glycosyltransferase enzyme complex, regulating *N‐*glycosylation modification at residue 263 of CTSD.

### 2.6 *N‐*glycosylation Modification of CTSD Regulates ACADM/Ferroptosis Axis to Promote CRC Progression

**Figure 6:**
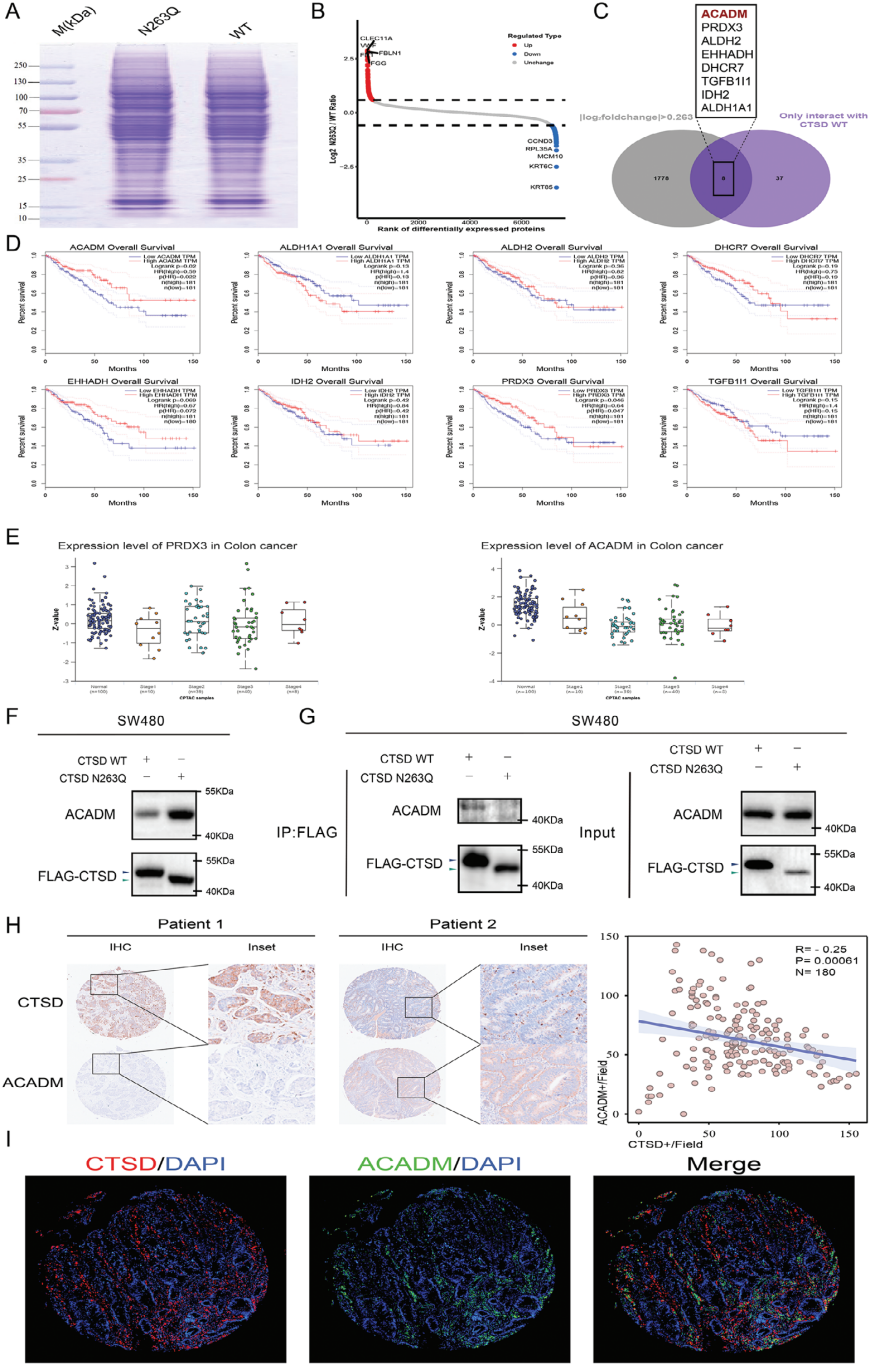
CTSD protease potentially degrades ACADM. A) Protein quality control staining charts. B) Differential protein abundance ranking scatter plot (*t*‐test, |log_2_ fold change| >.585, *p* < .05). C) Venn diagram illustrating intersecting proteins between differentially expressed proteins (|log_2_ fold change| > 0.263, *p* <0.05) and proteins enriched by CTSD WT. D) Survival analysis for 8 candidate molecules. Patients with high ACADM and PRDX3 expression demonstrated a more favorable prognosis than those with low ACADM and PRDX3 expression (*p* < .05). E) The CPTAC proteomics database was used to examine ACADM and PRDX3 protein levels in various stages of colon cancer and normal tissues. The expression levels of ACADM exhibited a decline concomitant with the progression of malignancy. Boxplots show median (central line), upper and lower quartiles (box limits), and 1.5 × interquartile range (whiskers). F) When CTSD WT and CTSD N263Q are expressed at equal levels, SW480 cells expressing CTSD N263Q exhibit a higher level of ACADM. G) Co‐immunoprecipitation analysis demonstrated that ACADM interacted with the CTSD WT, whereas no binding was observed with the CTSD N263Q variant. H) Immunohistochemical analysis showed that expression levels of ACADM were negatively correlated with expression levels of CTSD. I) Immunofluorescence analysis demonstrated the co‐localization of ACADM and CTSD.

To elucidate the potential downstream proteins influenced by *N‐*glycosylation modifications of CTSD, we conducted a comparative proteomic analysis between CTSD WT and the CTSD N263Q cells (**Figure** **6**A). We identified 391 differentially expressed proteins (DEPs, |log_2_ fold change| >0.263) between CTSD WT and CTSD N263Q cells (Figure 6B). Given the protease activity of CTSD, we conducted an intersection analysis between protein sets only interacting with CTSD WT and DEPs. This analysis yielded a total of 8 candidate molecules:ACADM, PRDX3, ALDH2, EHHADH, DHCR7, TGFB1I1, IDH2, and ALDH1A1 (Figure 6C).

In comparison to the normal group, the expression levels of *ACADM* and *ALDH1A1* were significantly down‐regulated in the tumor group, whereas the expression level of DHCR7 was significantly up‐regulated in the tumor group (Figure S2, Supporting Information). Kaplan–Meier survival analysis revealed that expression levels of *ACADM* and *PRDX3* were significantly associated with survival prognosis (Figure 6D). We investigated the link between candidate molecules and metastasis (TNM staging). Results indicated that expression levels of ACADM decreased as malignancy progressed (Figure 6E), suggesting a stronger association with liver metastasis (Stage 4). Therefore, ACADM was selected for the subsequent analysis.

When both CSTD WT and CTSD N263Q cells exhibited equivalent levels of CTSD expression, we observed that CTSD N263Q cells displayed higher levels of ACADM than CSTD WT cells (Figure 6F). Co‐immunoprecipitation analysis revealed a significant interaction between ACADM and CTSD WT, whereas no such interaction was observed with the CTSD N263Q variant (Figure 6G).

Immunohistochemical techniques were employed to assess the positive expression scores of CTSD and ACADM in 90 CRC tissues and corresponding adjacent normal tissues within the same visual field. The analysis revealed a negative correlation between expression levels of CTSD and ACADM (Spearman correlation, Figure 6H). Multiple immunofluorescence assays indicated the co‐localization of CTSD and ACADM (Figure 6I). These results further verify that *N‐*glycosylation modification at residue 263 of CTSD affects the proteolytic degradation of ACADM by the CTSD protease.

ACADM, an enzyme involved in fatty acid metabolism, does not directly regulate cellular proliferation, invasion, or metastasis.^[^ ^13^ ^]^ Therefore, we hypothesized that the regulation of ACADM expression via *N‐*glycosylation modification of CTSD at residue 263 may influence the invasiveness and metastasis of CRC cells through mechanisms associated with fatty acid metabolism. We utilized the WikiPathways dataset^[^ ^14^ ^]^ to conduct an enrichment analysis of DEPs between CTSD WT and CTSD N263Q cells. Our findings indicated that these DEPs were enriched in ferroptosis pathways related to lipid metabolism (WP4313 FERROPTOSIS) (Figure S3A, Supporting Information). Therefore, we hypothesized that *N‐*glycosylation modification of CTSD at residue 263 may influence the biological behaviors of CRC cells through the ACADM/ferroptosis axis.

We primarily analyzed expression levels of ferroptosis proteins ACSL4, SLC7A11, and GPX4. The expression levels of ferroptosis‐related proteins (SLC7A11 and GPX4) were lower in CTSD N263Q cells than in CTSD WT cells (Figure S3B, Supporting Information). Additionally, expression levels of ACADM exhibited a positive correlation with those of ACSL4, while demonstrating a negative correlation with the expression levels of SLC7A11 and GPX4 (Figure S3B, Supporting Information). Therefore, ACADM and *N‐*glycosylation modification of CTSD at residue 263 regulate the expression levels of ferroptosis‐related proteins, such as ACSL4, SLC7A11, and GPX4.

We also observed that the overexpression of ACADM inhibited the proliferation and invasion of SW480 and SW620 cells (Figure S3C−E, Supporting Information). Following the knockdown of the *N‐*glycosyltransferase STT3B and DDOST in CTSD, the invasive and proliferation capability of CTSD WT cells was significantly reduced, which was consistent with that observed in CTSD N263Q and CTSD N263Q/N134Q cells (Figure S3C−E, Supporting Information). These findings reaffirm that ACADM, STT3B, and DDOST modulate the biological behaviors of CRC cells through the regulation of CTSD *N‐*glycosylation. Therefore, CTSD, modulated by the *N*‐glycosyltransferases complex STT3B and DDOST through the addition of *N*‐glycosylation modification at residue 263, mediates the ACADM/ferroptosis‐related pathway axis and subsequently affects the invasive capacity of CRC cells.

## 3 Discussion

CRC poses a serious health threat globally.^[^ ^1^ ^]^ In patients with CRC, metastasis is the primary determinant of unfavorable prognosis, with hepatic metastasis being the most prevalent form.^[^ ^3^ ^]^ It is imperative to elucidate the molecular mechanisms underlying liver metastasis in CRC, with the aim of identifying novel therapeutic targets. Numerous studies have demonstrated that *N‐*glycosylation of proteins plays a significant role in various processes associated with malignant tumor metastasis.^[^ ^7^ ^]^ However, the differences in *N‐*glycosylated protein profiles between primary lesions and paired liver metastatic lesions in CRC remain unclear. In our study, we identified the differential expression profiles of *N‐*glycosylated proteins between primary lesions and paired liver metastatic lesions in 14 cases of CRC. This research offers a significant theoretical foundation for advancing the understanding of the molecular mechanisms underlying liver metastasis in CRC and for identifying novel therapeutic targets.

CTSD was initially linked to cancer in breast cancer,^[^ ^15^ ^]^ and overexpression of CTSD was shown to alter its processing, cell localization, and secretion.^[^ ^16^ ^]^ CTSD overexpression is now recognized as a marker of poor prognosis associated with clinical metastasis.^[^ ^17^ ^]^ The distinct *N‐*glycosylation structure of CTSD in tumor tissues and serum in patients with breast cancer relative to normal tissues^[^ ^18^ ^]^ suggests that varying *N‐*glycosylation patterns of CTSD may be closely linked to tumorigenesis. Our study found that increased *N‐*glycosylation modification of CTSD at residue 263 was significantly associated with liver metastasis in CRC and influenced the growth, proliferation, invasion, and metastasis of CRC cells. Therefore, this modification may be crucial for liver metastasis in CRC.

*N‐*glycosylation modification of CTSD appears to play distinct roles depending on the specific *N‐*glycosylation structures, species, or tissues involved.^[^ ^19^ ^]^ Previous studies have demonstrated that the *N‐*glycosylation modification of CTSD is essential for its proper localization to lysosomal compartments.^[^ ^19^, ^20^ ^]^ This finding is also corroborated by our observation that CTSD missing the *N*‐glycosylation modification at residue 263 or both 134/263 cannot co‐locate with endosomal/lysosomal markers in CRC. Previous studies have debated whether *N‐*glycosylation promotes CTSD secretion.^[^ ^21^ ^]^ Our findings in CRC revealed that losing *N‐*glycan at residue 263 hindered CTSD secretion, whereas losing *N‐*glycans at 2 sites increased its secretion. The underlying mechanism requires further investigation. *N‐*glycosylation modification has been reported to influence the extracellular stability of CTSD but not its intracellular stability.^[^ ^19c^ ^]^ However, in our studies, *N‐*glycosylation modification also affected the intracellular stability of CTSD. Previous studies have indicated that *N‐*glycosylation modification of CTSD had no effect on its enzymatic activity. However, utilizing the substrate sequence GKPILFFRLK(Dnp)‐D‐R‐NH2null, we found that *N‐*glycosylation modification did indeed affect the enzymatic activity of CTSD. This discrepancy in results may be attributed to the different enzyme substrates used, and warrants further investigation.

We also attempted to identify the *N‐*glycosylated oligosaccharide transferase (OST) of CTSD. *N‐*glycosylation is initiated by the oligosaccharide transferase complex in the endoplasmic reticulum. Our study found that *N‐*glycosylation modification at residue 263 of CTSD may be regulated by the OST complex protein DDOST, with the OST complex protein STT3B potentially serving as the catalytic subunit at the specific site. Furthermore, by modulating the expression levels of STT3B and DDOST, the impact of *N‐*glycosylation modification at residue 263 of CTSD on the biological behaviors of CRC cells can be replicated in vitro.

ACADM, a medium‐chain acyl‐CoA dehydrogenase, initiated β‐oxidation, an aerobic process by which fatty acids are degraded into acetyl‐CoA for energy production.^[^ ^22^ ^]^ ACADM has been identified as a tumor suppressor that inhibits tumorigenesis and tumor progression in liver and pancreatic cancers.^[^ ^23^ ^]^ Our study demonstrated that *N‐*glycosylation modification at the residue 263 of CTSD modulated the expression levels of ACADM and ferroptosis‐related proteins, including ACSL4, SLC7A11, and GPX4, thereby altering the biological behaviors of CRC cells.

Our study has several limitations. First, this was a single‐center study, and the CRC cohort exclusively comprised Chinese patients, which may introduce potential selection bias. Therefore, further prospective studies involving multi‐center cohorts and diverse ethnic populations are necessary to validate our findings. Second, we did not explore potential causes for discrepancies observed in comparison with prior studies. Lastly, a detailed investigation of the downstream regulatory factors influenced by *N‐*glycosylation modification at residue 263 of CTSD was not conducted. Future research should incorporate additional analyses and experiments to elucidate the precise roles of the *N*‐glycosylation modification of CSTD, and the underlying mechanisms, in liver metastasis in CRC.

In conclusion, *N‐*glycosylation modification at residue 263 of CTSD has the potential to alter liver metastasis in CRC.

## 4 Conclusion

In this study, we analyzed the *N‐*glycosylation modification protein expression profiles in primary lesions and paired liver metastatic lesions of CRC by Liquid Chromatography ‐Mass Spectroscopy (LC‐MS) analysis. *N‐*glycosylation modification at residue 263 of CTSD affects liver metastasis in CRC. This modification, regulated by the glycosyltransferases complex DDOST and STT3B, affects the cellular localization, stability, and enzymatic activity of CTSD. Furthermore, it influences the invasion and metastasis of CRC cells by modulating the ferroptosis pathway mediated by ACADM. These findings may provide potential therapeutic targets for CRC treatment as well as strategies for controlling CRC progression and metastasis.

## 5 Experimental Section

### Clinical Sample Acquisition

This study was approved by the Research Ethics Committee of Peking University People's Hospital (approval number: 2021PHB267‐001). Prior to sample collection, written informed consent was obtained from all participants. The clinicopathological characteristics of included patients are presented in Table S4 (Supplementary Table).

Patients who received surgical treatment at the Department of Gastrointestinal Surgery, Peking University People's Hospital, between November 2019 and July 2022, and were subsequently pathologically diagnosed with liver metastasis secondary to synchronous CRC, were selected. Samples from primary lesions and paired liver metastatic lesions were collected. Cases with incomplete clinicopathological data or those that failed quality inspection following total protein extraction were excluded. Additionally, cases presenting with intestinal obstruction, tumor‐induced intestinal perforation, or cancer thrombus formation during treatment were excluded.

### Cell Lines

293T, NCM460, SW480, SW620, RKO, DLD1, LoVo, HCT8, and HCT116 cell lines were obtained from the Cell Resource Center of the Chinese Academy of Sciences. Cells were cultured in 10% fetal bovine serum (Gibco, USA) and 1% penicillin‐streptomycin (Gibco, USA), and incubated at 37 °C and 5% CO2 in an incubator.

### LC‐MS Analysis for Glycopeptide—Glycopeptide Enrichment and Proteolysis

Primary lesions and paired liver metastatic lesions from patients were cut into pieces, and ground in liquid nitrogen using a mortar and pestle until the tissues became mucilaginous. Radio Immunoprecipitation Assay lysis buffer was added to the tissue samples to form a homogeneous solution, and samples were incubated for 30 min. The samples were then centrifuged at 12 000 g for 20 min, and the supernatant was transferred to ice. The concentration of extracted proteins was measured using the Pierce BCA Protein Assay which is an approach with high‐precision and detergent‐compatible.

The extracted proteins were incubated with 10 mM DTT at 37 °C, for 1 h, to reduce the disulfide bonds in the glycoproteins. To avoid regeneration of disulfide bonds, carboxymethylation of sulfhydryl groups was subsequently performed by incubating the samples in 20 mM IAM at room temperature in the dark for 2 h. Redundant chemicals were eliminated before tryptic digestion using 3K ultrafiltration tubes. Then, the glycoproteins were digested with trypsin at a ratio of 50:1 (w/w). Subsequently, the glycopeptides were purified and enriched using a hydrophilic HILIC solid phase extraction (SPE) column. Briefly, the tryptic peptides were dissolved in 80% ACN and 1% TFA. Before sample‐loading, the HILIC SPE columns were washed twice with 0.1% TFA and 80% ACN and 1% TFA. After samples were loaded, the columns were washed twice with 0.5 mL 80% ACN and 1% TFA. The glycopeptides bound to the columns were eluted twice with 0.5 mL 0.15%TFA solution. The eluted glycopeptides were dried via vacuum centrifugation, and stored at −80 °C for later use.

### LC‐MS Analysis of Intact N‐glycopeptides

Intact *N*‐linked glycopeptide samples were analyzed using the Orbitrap Eclipse Tribrid mass spectrometer (Thermo Fisher, USA). Glycopeptides were dissolved in 0.1% formic acid (buffer A). Then, samples were separated using the C18 column (homemade), and the mobile phase flow rate was 300 nL mi^−1^n which consisted of 0.1% FA in water (A) and 80% ACN/0.1% FA (B). Samples were separated in a single LC‐MS run using a 60‐min gradient, and the gradient profile for LC separation was set as follows: 5%–8% buffer B, 0–10 min; 8%–22% buffer B, 10–34 min; 22%–32% buffer B, 34–48 min; 32%–90% buffer B, 48–49 min; 90% buffer B, 49–60 min.

The Orbitrap MS^1^ spectra were collected from 350–2000 m z^−1^ at a resolution of 60 000, and the spray voltage was set at 2000 V in the positive mode. A dynamic exclusion time of 20 s was used to discriminate against previously selected ions. Data‐dependent HCD MS^n^ (*n* ≥ 2) scanning was performed at the mass range of 120–2000 m z^−1^ using an isolation window of 1.6 m z^−1^ at a resolution of 30 000. The collision energy was set at 20%‐40%, and charge state screening was enabled to reject unassigned and singly charged ions. Tune application (Thermo Fisher Scientific) and Xcalibur software (Thermo Fisher Scientific) were used for instrument control and mass spectrometry data collection.

### Identification and Quantification of N‐Glycopeptides

Identification of intact *N‐*glycopeptides was performed using pGlyco 3.0 and our previously developed intelligent glycan identification strategy, GIPS.^[^ ^24^ ^]^ Peptide sequences, *N‐*glycosylation sites, and glycan components were analyzed using pGlyco 3.0. Briefly, raw data were directly fed into pGlyco 3.0, and the database of human proteins extracted from UniProt was searched. The flow type was set as *N‐*linked, and trypsin was set as enzymatic digestion with up to 2 missed cleavages. The carbamidomethylation was set as dynamic modification, and the mass tolerance for the precursor and fragment ions were defined as ±5 and ±20 ppm, respectively. The *N‐*glycosylation sequence motifs (N‐X‐S/T, X ≠ P) were modified by switching “N” to “J” (both amino acids with the same mass). A quality control method for the identification of *N*‐linked glycopeptides was established, and the false discovery rate (FDR) for glycopeptide spectrum matching was set at 1%. The peptide sequence identified using pGlyco 3.0 was considered as fixed modifications of glycans, and the identification of glycan structures was carried out using the intelligent GIPS approach. Briefly, the GIPS approach was used to simulate fragments of all the isomeric glycan structures and to calculate the probability of isomeric glycan structures being the actual glycan structure using the integrated algorithm. The resulting files from pGlyco 3.0 were subsequently imported into pGlycoQuant for quantification of identified *N‐*glycopeptides.^[^ ^25^ ^]^

### Identification of Differential Expression N‐glycopeptides

Differentially expressed glycopeptides were identified by the Clinical Medical Statistical Analysis Platform (http://msap.cqxyy.org.cn/login?redirect = %2Findex). Briefly, we first performed a normal distribution test and homogeneity test of variance of each *N‐*glycopeptide, and then performed a *t*‐test on glycopeptides satisfying these 2 tests. Otherwise, a nonparametric test was accordingly conducted. Mann‐Whitney test was performed for a 2‐group test. The results were subsequently visualized through an online platform (https://www.bioinformatics.com.cn/) based on an “EnhancedVolcano” R package. Glycopeptides exhibiting |log_2_ fold change| > .585 and *p* < .05 were classified as differentially expressed glycopeptides.

### Enrichment Analysis of N‐Glycosylated Proteins

UniProt (http://www.uniprot.org/) was utilized to identify the gene name corresponding to *N*‐glycosylated proteins. These genes were subjected to Gene Ontology (GO) and Kyoto Encyclopedia of Genes and Genomes (KEGG) pathway enrichment analysis in DAVID (https://david.ncifcrf.gov/) dataset. Fisher's exact test was applied to identify the significant enrichment GO categories and KEGG pathways of these genes, and FDR was used to correct p values (*p* < .05). The results were subsequently visualized utilizing an online platform (https://www.bioinformatics.com.cn/).

### Western Blot Assay

The methodology was consistent with previous procedures.^[^ ^26^ ^]^ Anti‐rabbit CTSD (AB‐75811, Abcam), Anti‐mouse CTSD (AB‐302649, Abcam), Anti‐rabbit CTSD (AB‐75852, Abcam), Anti‐mouse GAPDH (60004‐1‐Ig, Proteintech), Anti‐rabbit IgG, HRP‐linked Antibody (Cell Signaling Technology), Anti‐mouse IgG, HRP‐linked Antibody (Cell Signaling Technology), Anti‐mouse ACADM (67742‐1‐Ig, Proteintech), Anti‐rabbit DDOST (14916‐1‐AP, Proteintech), Anti‐mouse STT3A (66581‐1‐Ig, Proteintech), Anti‐mouse STT3B (15323‐1‐AP, Proteintech), Anti‐rabbit ACSL4 (AB‐155282, Abcam), Anti‐rabbit SLC7A11 (AB‐307601, Abcam) and Anti‐rabbit GPX4 (AB‐125066, Abcam) were used.

### PNGase F Processing

A total of 20 µg of extracted cellular proteins was combined with 1 µL of 10× Glycoprotein Denaturing Buffer, and deionized water was added to achieve a final volume of 10 µL. The mixture was then subjected to centrifugation and incubated at 100 °C for 10 min. Following incubation, the denatured sample was promptly placed on ice and rapidly centrifuged. Subsequently, 2 µL of 10× Glyco Buffer 2 (Yi Sheng, China), 2 µL of 10% NP‐40 (Yi Sheng, China), 1 µL of PNGase F (Yi Sheng, China), and 5 µL of deionized water were added to the sample. The resulting mixture was centrifuged and incubated at 37 °C for 1 h.

### Tunicamycin Processing

Tunicamycin powder (MCE, USA) was dissolved in DMSO to prepare a drug solution with a concentration of 2 µg µL^−1^. This solution was subsequently added to the experimental medium to achieve a final concentration of 2 µg mL^−1^. An equivalent volume of DMSO was added to the control group. Total proteins were extracted from the lysed cells 24 h post‐treatment for subsequent analysis.

### Site‐Directed Mutagenesis

The coding DNA sequence for *CTSD* was retrieved from the NCBI database (http://www.ncbi.nlm.nih.gov/) and the codon sequence corresponding to the amino acid site to be mutated was determined. The Asparagine (N) residue was substituted with Glutamine (Q), chosen for its closely related physicochemical properties; accordingly, the codon was altered from AAT to CAG. Upstream primers were designed by selecting the sequence comprising 11–13 bases upstream and downstream of the mutation site, and the mutation site itself. The reverse complementary sequence of the upstream primers was utilized as the downstream primers. Plasmid DNA was isolated by PCR and agarose gel electrophoresis. The CTSD mutant constructs were subsequently verified through sequencing and used in further experimental procedures.

### Plasmid Construction

The CTSD shRNA was specifically engineered to target the 3′ untranslated region (UTR) of CTSD mRNA, ensuring that it did not interfere with the expression of the exogenous CTSD plasmid. Puromycin resistance cassettes were used for the lentiviral plasmid of CTSD shRNA, and Blasticidin resistance cassettes were used for the lentiviral plasmid of CTSD WT, CTSDN263Q, and CTSDN263Q∖N134Q. To meet the requirements of subsequent experiments, all plasmids were designed to be non‐fluorescent.

### Cell Transfection/Lentiviral Infection

Lipofectamine 3000 (Invitrogen, USA) was used for transient transfection. The CTSD shRNA lentiviral vector was utilized to silence CTSD expression in CRC cells. Subsequently, CTSD WT, CTSD N263Q, and CTSD N263Q/N134Q lentiviruses were transfected into these CTSD‐silenced CRC cell lines to generate new cell lines that stably expressed CTSD WT, CTSD N263Q, and CTSD N263Q/N134Q proteins, respectively.

### Cell Proliferation, Migration, and Invasion Assays

These assays were performed in accordance with previous procedures.^[^ ^26^ ^]^

### Flow Cytometry Assay

A 2× EdU working solution and Click Additive Solution were prepared according to the manufacturer's instructions (Biyuntian Biology, China). Cells in the logarithmic growth phase were selected. An equal volume of preheated 2× EdU working solution (20 µM) at 37 °C was added to each well of a 6‐well plate. The cells were incubated for 45 min. Subsequently, cell proliferation was assessed by flow cytometry following a series of steps including fixation, washing, permeabilization, and the Click reaction with paraformaldehyde.

Then, 1× Annexin binding buffer was prepared in accordance with the manufacturer's instructions (Union Biology, China). The cells were resuspended in 100 µL of the formulated 1× Annexin binding buffer. Subsequently, 5 µL of Alexa Fluor 488 Annexin V and 1 µL of 100 µg mL^−1^ propidium iodide (PI) were added to each tube. The samples were incubated at room temperature for 15 min, protected from light. Following incubation, 400 µL of the 1× Annexin binding buffer was added to each tube. Apoptosis was then assessed by flow cytometry.

The cell cycle was analyzed using a cell cycle staining kit (Biobiology, China). Cells in the logarithmic growth phase were treated with 1 mL of DNA Staining Solution and 10 µL of Permeabilization Solution. The mixture was subjected to vortex oscillation for 5–10 s. Subsequently, the cells were incubated at room temperature for 30 min in the absence of light. Flow cytometry was employed to measure the cell cycle.

### Nude Mouse Subcutaneous Tumor Formation and Liver Metastasis Model

For subcutaneous tumor formation in nude mice, female Balb/c nude mice, aged 4–6 weeks, were randomly assigned to one of 2 groups—CTSD WT group and the CTSD N263Q group—based on the types of implanted cells. Then, 100 µL of the cell suspension, containing 5 × 10^5^ cells, was injected into the right dorsal subcutaneous region of nude mice. Post‐inoculation, tumor formation was monitored every 3 days. Upon macroscopic detection of the tumor, the longest and perpendicular diameters were measured using a vernier caliper. Tumor volume was calculated using the formula V = 0.5 × L (length) × W^2 (width). A tumor growth curve was then plotted based on these measurements.

Next, a liver metastasis model was established with the following protocol. The cell concentration was adjusted to 2×10^7^cells mL^−1^ using the aforementioned method. Cell suspensions of ≈50 µL, containing 1 × 10^6^ cells, were injected into the spleen using a 1 mL syringe. Four weeks post‐injection, the nude mice were euthanized to assess tumor metastasis in the spleen, liver, and lungs. The number of metastatic sites was quantified and represented as a histogram. Liver tissues were subsequently fixed with paraformaldehyde, followed by paraffin embedding and sectioning for further analysis.

### Immunofluorescence

The cell culture dish was pre‐coated with climbing tablets, and the cells were cultured overnight. Following cell adhesion, the cells were fixed using paraformaldehyde, permeabilized with Triton X‐100, blocked with bovine serum albumin (BSA), incubated with primary and secondary antibodies, counterstained with DAPI tablets, and subsequently observed under a fluorescence microscope. The following antibodies were used: Anti‐rabbit LAMP1 (Catalog Number 9091, Cell Signaling Technology), Anti‐rabbit EEA1 (Catalog Number 3288, Cell Signaling Technology), Anti‐rabbit Rab7 (Catalog Number 9367, Cell Signaling Technology), 555‐conjugated secondary antibody (Abcam, UK) and 647‐conjugated secondary antibody (Abcam, UK).

### Enzyme Functional Activity Detection

The Cathepsin D Activity Assay Kit (Abcam) was used with the preferred CTSD substrate sequence, GKPILFFRLK(Dnp)‐D‐R‐NH2, labeled with MCA. Upon incubation with a cell lysate containing CTSD, the substrate was cleaved, resulting in the release of fluorescence. This fluorescence was subsequently quantified using an enzymometer, with excitation and emission wavelengths set at 328 and 460 nm, respectively.

### LC‐MS Analysis for Protein‐CTSD Enrichment

Initially, total proteins were extracted from both the CTSD WT stable strain and the CTSD N263Q stable strain of SW480 cells. Subsequently, the CTSD protein, tagged with a FLAG label, was enriched using FLAG magnetic beads. Following the treatment of equal amounts of protein samples, the samples were subjected to electrophoresis, fixation, sensitization, and silver staining. Following the dyeing of the samples, images of the silver‐stained gels were captured.

### Collecting Total Proteins

Similarly, total proteins were extracted from both the CTSD WT stable strain and the CTSD N263Q stable strain of SW480 cells. Equal amounts of protein samples were subjected to electrophoresis, followed by Coomassie Brilliant Blue staining, decolorization, and additional procedures. Images of the stained gels were used to document the results.

### LC‐MS Analysis of Proteins

The peptides were solubilized using the mobile phase A of liquid chromatography, which consisted of an aqueous solution containing 0.1% formic acid and 2% acetonitrile. Separation was performed using the EASY‐nLC 1200 Ultra‐High‐Performance Liquid Chromatography system (Thermo Fisher, USA). The liquid phase gradient was programmed as follows: 0–22.5 min, 6%–22% buffer B (comprising an aqueous solution of 0.1% formic acid and 90% acetonitrile); 22.5–26.5 min, 22%–34% buffer B; 26.5–28.5 min, 34%–80% buffer B; 28.5–30 min, 80% buffer B, the flow rate was consistently maintained at 700 nL min^−1^. The peptides were fractionated using an ultra‐high‐performance liquid chromatography system and subsequently introduced into a nano‐spray ionization source for ionization. The ionized peptides were then subjected to analysis via Orbitrap Exploris 480 mass spectrometry. The ion source voltage was maintained at 2300 V, while the FAIMS (Field Asymmetric Ion Mobility Spectrometry) compensation voltage was configured to −45 V. High‐resolution Orbitrap mass spectrometry was performed to detect and analyze the peptide precursor ions along with their secondary fragments. The first mass spectrometry was configured with a scanning range of 350–1400 m z^−1^ and a resolution of 60000. In contrast, the secondary mass spectrometry was set to a fixed scanning range of 120 m z^−1^ with a resolution of 15000. Data acquisition was conducted using a data‐independent acquisition (DIA) method. In this procedure, peptide ions were sequentially introduced into the higher‐energy collisional dissociation collision cell through multiple successive m/z windows following the first scan, where they were fragmented using 27% fragmentation energy, subsequently undergoing secondary mass spectrometry analysis in turn. To enhance the efficient utilization of the mass spectrum, the automatic gain control (AGC) was configured to 1E6, and the maximum injection time was adjusted to 22 ms.

### Identification and Quantification of Proteins

The DIA data were processed using the DIA‐NN search engine (version 1.8) and using the software's default parameters. The database utilized was Homo_sapiens_9606_SP_20 230 103.fasta, comprising 20389 sequences. The enzymatic cleavage method employed was Trypsin/P, with the maximum allowable number of missed cleavages set to one. Fixed modifications were specified as N‐terminal methionine excision and cysteine carbamidomethylation. The theoretical spectral library was generated using a deep learning algorithm, with an inverse library incorporated to assess the FDR attributable to random matching. The FDR threshold for precursor identification was established at 1%. To ensure high‐quality analytical outcomes, the results from the database search require further filtering. The filtering criteria for the identification results: a precursor and protein with FDR of 1%; each identified protein with at least one unique peptide.

### Identification and Enrichment Analysis of Differential Expression Proteins (DEP)

The “DEP” R package, in conjunction with Benjamini‐Hochberg‐adjusted p values, was employed to identify DEPs between CTSD WT and CTSD N263Q cells (*t*‐test, |log_2_ fold change| > .263, *p* < .05), and the results were subsequently visualized through “ggplot2” R package. We used the WikiPathways dataset (https://www.wikipathways.org/) to annotate enrichment pathways of DEPs between CTSD WT and CTSD N263Q cells. Fisher's exact test was applied to identify the significant WikiPathways pathways, and FDR was used to correct *p* values. The results were subsequently visualized utilizing an online platform (https://www.bioinformatics.com.cn/).

### Co‐Immunoprecipitation Analysis (co‐IP)

Total protein extraction was extracted using BC100 buffer, which comprised 100 mmol L^−1^ sodium chloride, 20% glycerol, 0.2% NP40, 50 mmol L^−1^ Tris, 1 mmol L^−1^ DTT, and 1 mmol L^−1^ PMSF, with the pH adjusted to 7.9 using HCl. The protein concentration was subsequently quantified. The target protein was then captured at low temperatures using a FLAG affinity gel (A220, Sigma Aldrich), followed by a purification step to remove impurities. Competitive elution of the target protein was achieved using the FLAG peptide (Biyuntian Biological, China). Finally, the purified protein was analyzed using mass spectrometry or western blot.

### Gene and Protein Expression Level Analysis and Survival Analysis

We investigated gene expression levels (log2(TPM+1)) of Colon adenocarcinoma (COAD, *n* = 275), Rectum adenocarcinoma (READ, *n* = 92) patients and normal control (51) utilizing an online platform ^[^ ^10^ ^]^ (http://gepia.cancer‐pku.cn/index.html) where it based on the TCGA datasets, used One–way analysis of variance (ANOVA) to calculating differential expression between tumor (COAD and READ) and normal control. Patients with CRC (*n* = 181) were stratified into 2 cohorts characterized by high and low gene expression levels, determined according to the median gene expression value. Overall survival curves for these 2 groups were generated utilizing the online platform (http://gepia.cancer‐pku.cn/index.html) based on the Kaplan‐Meier analysis (log‐rank test). Additionally, we utilized the UALCAN online platform (https://ualcan.path.uab.edu/), which is based on the Clinical Proteomic Tumor Analysis Consortium (CPTAC) database (https://proteomics.cancer.gov/programs/cptac), to analyze protein expression levels across various stages in patients with CRC. Z‐values represent standard deviations from the median across samples for CRC samples. Log2 spectral count ratio values from CPTAC were first normalized within each sample profile and then normalized across samples.

### Immunohistochemistry

The paraffin‐embedded sections were subjected to a series of preparatory steps, including dewaxing to water, antigen retrieval, blocking of endogenous enzymes, preparation of slides, sealing to prevent non‐specific binding, incubation with primary and secondary antibodies, DAB chromogenic development, nuclear counterstaining, sealing for preservation, and microscopic evaluation. These procedures ensured optimal antigen exposure and antibody binding. Subsequently, the images were analyzed and quantified using microscopic observation and ImageJ software (https://imagej.net/ij/index.html). Spearman's rank correlation was used to analyze the correlation between CTSD and ACADM.

### Statistical Analysis

The flowchart figure was drawn using the Figdraw platform (https://www.figdraw.com/). Mann‐Whitney test was performed to identify differential expression glycopeptide. The Benjamini‐Hochberg‐adjusted P values of DEPs were calculated in R (version 4.1.3, https://www.r‐project.org/). Fisher's exact test was employed for the enrichment analysis of categorical data. Experimental statistical data were analyzed and visualized using GraphPad Prism software (version 9, https://www.graphpad.com/features), and continuous variables from 2 independent samples were assessed using the *t*‐test. The log‐rank test was employed to evaluate the statistical significance of differences between Kaplan‐Meier survival curves. One‐way analysis of variance (ANOVA) was used to evaluate the differences in gene expression between the 2 groups. The association between 2 groups was analyzed utilizing Spearman's rank correlation. Each group underwent 3 independent experiments.

## Conflict of Interest

The authors declare no conflict of interest.

## Author Contributions

N.X., Y.D., C.H. contributed equally to this work. N.X. contributed data curation, formal analysis, methodology, resources, software, validation, and visualization, and wrote, reviewed, and edited the original draft. Y.D. contributed data curation, formal analysis, methodology, resources, software, validation, and visualization. C.H. contributed data curation, formal analysis, methodology, resources, validation, and visualization, and wrote, reviewed, and edited the original draft. L.Z., C.Y., Q.S., Z.G., C.W., J.Z., H.Z., and S.W. contributed methodology, software, validation, and visualization. Y.Y. contributed data curation, formal analysis, methodology, resources, and supervision. Y.L. contributed data curation, formal analysis, funding acquisition, methodology, resources, and supervision, and wrote, reviewed, and edited the original draft. Z.S. contributed data curation, formal analysis, funding acquisition, methodology, resources, supervision, validation, and visualization, and wrote, reviewed, and edited the original draft.

## Supporting information



Supporting Information

Supplementary Table

Supplementary Table

Supplementary Table

Supplementary Table

## Data Availability

The data that support the findings of this study are available in the supplementary material of this article.
