# Supplementary material for: N‐glycosylation Modification of CTSD Affects Liver Metastases in Colorectal Cancer
Source: Adv Sci (Weinh). 2024 Dec 24;12(7):2411740. doi: 10.1002/advs.202411740 (PMC11831497; doi:10.1002/advs.202411740)
Supplement: Supplementary file 1 — Supporting Information [file ADVS-12-2411740-s005.docx]

*N-*glycosylation Modification of CTSD Affects Liver Metastases

**in Colorectal Cancer**

*Nan Xiong,^1,2,3#^ Yan Du,^1,2,3#^ Chuncui Huang,^4,5#^ Quanyi Yan,^6^ Long Zhao,^1,2,3^ Changjiang Yang,^1,2,3^ Qing Sun,^4,5^ Zhidong Gao,^1,2,3^ Caihong Wang**,^1,2,3^ Jun Zhan,^7^ Hongquan Zhang,^7^ Shan Wang,^1,2,3^ Yingjiang Ye,^1,2,3*^ Yan Li,* *^4,5^* Zhanlong Shen**,^1,2,3*^*

^1^Department of Gastroenterological Surgery, Peking University People's Hospital, Beijing, 100044, China

^2^Beijing Key Laboratory of Colorectal Cancer Diagnosis and Treatment Research, Beijing, 100044, China

^3^Laboratory of Surgical Oncology, Peking University People's Hospital, Beijing, 100044, China

^4^Key Laboratory of Epigenetic Regulation and Intervention, Institute of Biophysics, Chinese Academy of Sciences, 15 Datun Road, Beijing 100101, China

^5^University of Chinese Academy of Sciences, 19 Yuquan Road, Beijing 100049, China

^6^Western Institute of Health Data Science, 28 High Tech Avenue, Chongqing 401329, China

^7^Program for Cancer and Cell Biology, Department of Human Anatomy, Histology and Embryology, School of Basic Medical Sciences, Peking University Health Science Center, Beijing, 100191, China

#These authors contributed equally.

*Correspondence:

Zhanglong Shen

[shenzhanlong@pkuph.edu.cn](mailto:shenzhanlong@pkuph.edu.cn)

Yan Li

[yanli@ibp.ac.cn](mailto:yanli@ibp.ac.cn)

Yingjiang Ye

[yeyingjiang@pkuph.edu.cn](mailto:yeyingjiang@pkuph.edu.cn)

**Supplementary Material**

**Figure S1 *N-*glycosylation modification of CTSD affected biological processes in colorectal cancer cells in vitro**

**(A)** Transwell assay results showed that the invasion ability of CTSD N263Q and CTSD N134Q/N263Q cells was significantly inhibited relative to to CTSD WT cells (n = 3; t-test; *p < 0.05, **p < 0.01 and ***p < 0.001; mean ± SEM; each group underwent three independent experiments). **(B)** The Colony formation ability of CTSD N263Q and CTSD N134Q/N263Q cells was obviously inhibited relative CTSD WT cells. (n = 3; t-test; *p < 0.05, **p < 0.01 and ***p < 0.001; mean ± SEM; each group underwent three independent experiments). **(C)** Flow cytometric analysis revealed a marked inhibition in proliferation rates of CTSD N134Q/N263Q cells relative to CTSD WT cells. **(D)** Annexin V and propidium iodide (PI) were used to detected apoptotic cells. Q3 represents the Annexin V+/PI- cells, early apoptotic cells. Flow cytometric analysis revealed that, in comparison with CTSD WT cells, the proportion of early apoptotic cells among both CTSD N263Q and CTSD N134Q/N263Q cells was increased, indicating suppression of the anti-apoptotic capacity in both CTSD N263Q and CTSD N134Q/N263Q cells. **(E)** Cell Counting Kit-8 analysis revealed the proliferation ability of CTSD N263Q and CTSD N134Q/N263Q cells was significantly inhibited relative to CTSD WT cells (n = 3; t-test; *p < 0.05, **p < 0.01 and ***p < 0.001; mean ± SEM; each group underwent three independent experiments). **(F)** Flow cytometric analysis revealed that the proportion of G2-M phase cells was increased in both CTSD N263Q and CTSD N134Q/N263Q cells was increased relative to CTSD WT cells, indicating suppression of proliferation capacity. (P3, G0-G1 phase; P4, S phase; P5, G2-M phase)

**
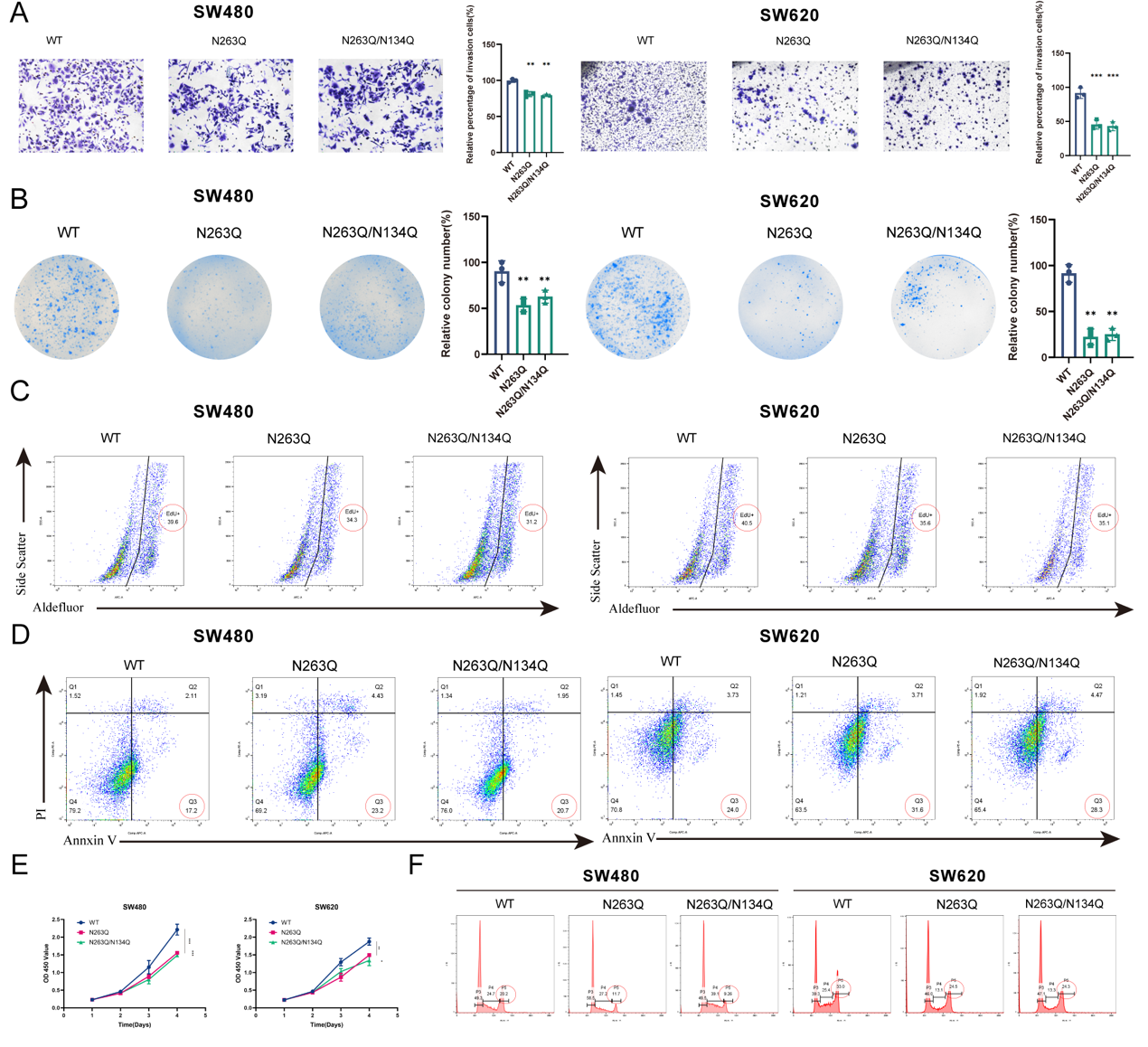
**

**Figure S2 Gene expression of candidate molecules in colorectal cancer and normal patients.**

Expression levels of eight candidate molecules in CRC patients and normal control (one-way ANOVA, *p < 0.05, Boxplots show median (central line), upper and lower quartiles (box limits), and 1.5 × interquartile range (whiskers)).

**
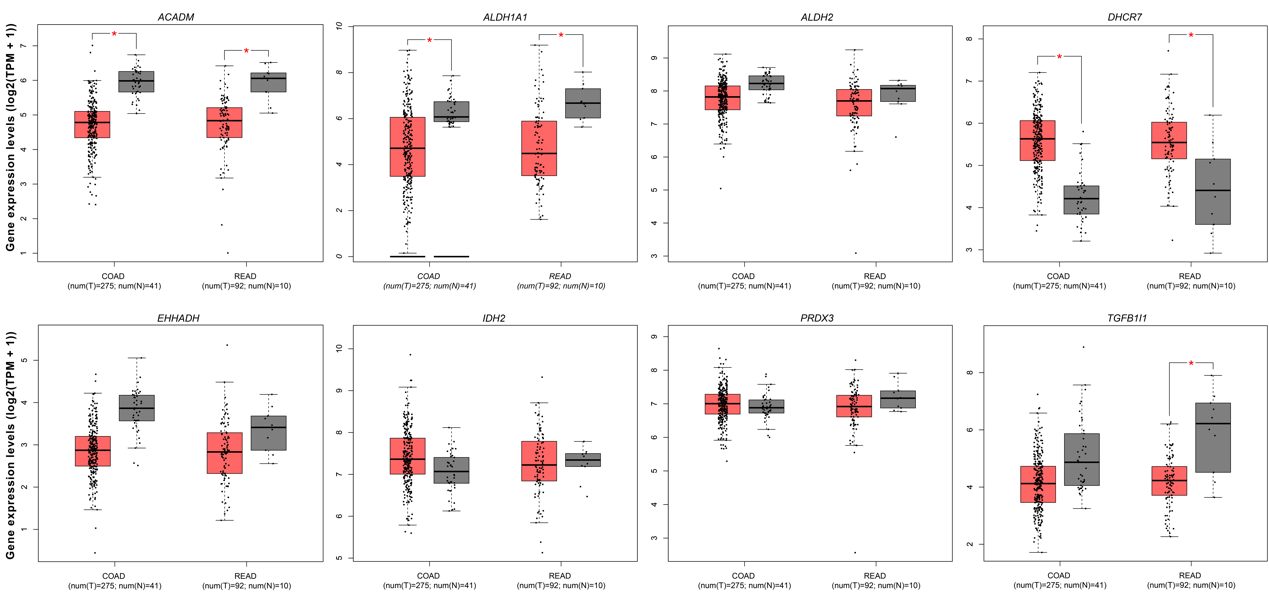
**

**Figure S3 ACADM, STT3B and DDOST affected biological processes in colorectal cancer (CRC) cells**

**(A)** Identifying enrichment pathways of differentially expressed proteins between CTSD WT and CTSD N263Q cells using the WikiPathways dataset (Fisher's exact test).

**(B)** Expression levels of ACADM and *N*-glycosylated modification at residue 263 of CTSD affected the expression levels of ferroptosis-related proteins, such as ACSL4, SLC7A11, and GPX4.

**(C)** Overexpression of ACADM and the knockdown of STT3B and DDOST significantly inhibited the invasion ability of CRC cells. (transwell assay; n = 3; t-test; *p < 0.05, **p < 0.01 and ***p < 0.001; mean ± SEM; each group underwent three independent experiments)

**(D)** Overexpression of ACADM and knockdown of STT3B and DDOST significantly inhibited colony formation by CRC cells. (n = 3; t-test; *p < 0.05, **p < 0.01 and ***p < 0.001; mean ± SEM; each group underwent three independent experiments)

**(E)** Overexpression of ACADM and the knockdown of STT3B and DDOST significantly inhibited the proliferation ability of CRC cells. (Cell Counting Kit-8 analysis; n = 3; t-test; *p < 0.05, **p < 0.01 and ***p < 0.001; mean ± SEM; each group underwent three independent experiments)

**
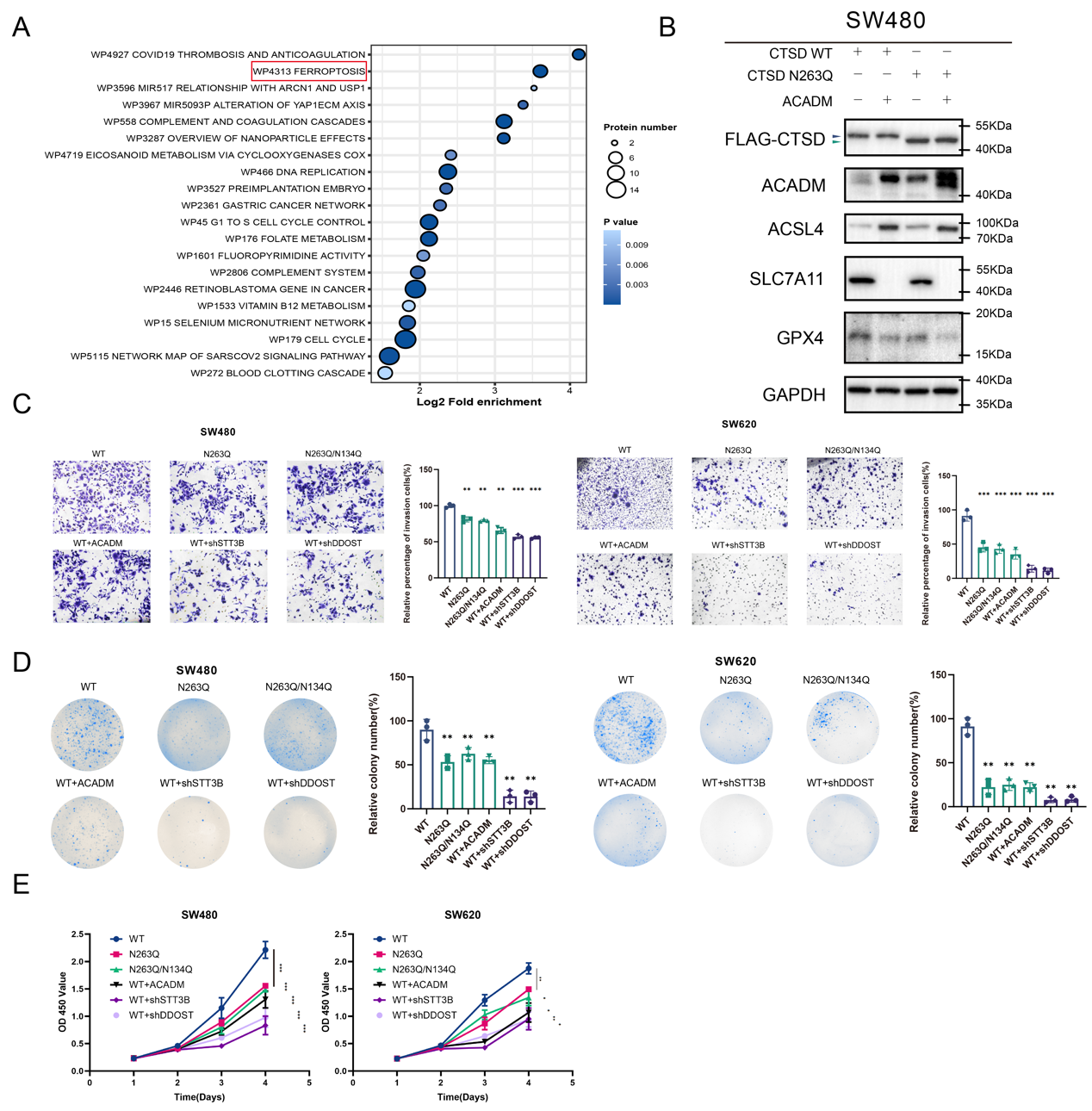
**

**Table S1** *N-*glycosylated protein profiles of primary lesions and paired liver metastatic lesions

**Table S2** *N-*glycosylation sites have been not reported by the Uniprot dataset

**Table S3** *N-*glycosylated modification proteins and sites of differential expression in primary and paired liver metastatic lesions

**Table S4** The clinicopathological characteristics of 14 patients with colorectal cancer
